# The Utility of Ultrasound in the Preoperative Localization of Primary Hyperparathyroidism: Insights from Pakistan

**DOI:** 10.7759/cureus.9835

**Published:** 2020-08-18

**Authors:** Tehseen Fatima, Bhagwan Das, Saadia Sattar, Sumerah Jabeen, Abid Abbas Khan, Najmul Islam

**Affiliations:** 1 Internal Medicine: Diabetes, Endocrinology and Metabolism, Aga Khan University Hospital, Karachi, PAK; 2 Internal Medicine, Aga Khan University Hospital, Karachi, PAK; 3 Internal Medicine: Diabetes, Endocrinology and Metabolism, Patel Hospital, Karachi, PAK; 4 Cardiology, Civil Hospital Dow University of Health Sciences, Karachi, PAK

**Keywords:** primary hyperparathyroidism, 99m-tc sestamibi scintigraphy, ultrasound neck, preoperative localization

## Abstract

Objective

We aimed to evaluate and compare the diagnostic performance of ultrasound (US) and 99m-Tc sestamibi scintigraphy for the preoperative localization of primary hyperparathyroidism (PHPT).

Methods

This retrospective study was conducted at the Aga Khan University Hospital in Karachi, Pakistan, and comprised the data of patients with PHPT who underwent parathyroidectomy from 2008 to 2017. Preoperative US and 99m-Tc sestamibi scintigraphy findings were recorded and compared to surgical and histological findings, which were taken as a reference standard.

Results

The sensitivity of US in the preoperative localization of PHPT was 88.3%, positive predictive value (PPV) was 94.6%, and accuracy was 84.1%. The sensitivity of 99m-Tc sestamibi scintigraphy was 90.4%, PPV was 94.3%, and accuracy was 85.7%.

Conclusion

US neck is an efficient tool for the preoperative localization of PHPT, demonstrating a comparable diagnostic yield with 99m-Tc sestamibi, and can serve as a credible first-line imaging modality in a resource-constrained healthcare setup.

## Introduction

Primary hyperparathyroidism (PHPT) is characterized by an autonomous dysregulated secretion of parathyroid hormone (PTH) from the parathyroid glands. The culprit lesion, in most cases, is an adenoma followed by multiple gland hyperplasia and, in rare situations, parathyroid carcinoma [1]. Surgical resection is the only definitive and standard treatment for PHPT [2]. Till the early 1990s, open neck exploration was the usual surgical technique implemented for parathyroid surgeries. However, for the last two decades, efforts at making the surgery least invasive and reducing the intraoperative and postoperative complications have led to the evolution of newer surgical methods that are collectively labeled as minimally invasive surgery of the parathyroid glands (MIP) [3]. With the increasing cosmetic concerns of the patients and the surgeons’ preference for minimal surgical trauma and duration, the application of MIP in modern-day practice is on the rise [4-5].

MIP includes a number of procedures and approaches devised to reduce operative duration, surgical trauma, and postoperative complications, and achieve improved cosmetic results [6]. Studies have shown the superiority of MIP over open neck exploration in terms of shortened hospital stay, lower complication rates and reduced healthcare-related cost, and better cosmetic satisfaction for the patients [7]. The success of surgery, however, is largely dependent on the preoperative localization of the parathyroid pathology, for both MIP as well as open neck exploration.

Several imaging modalities have been employed worldwide for this purpose, including ultrasonography (US), MRI, 99m-Tc sestamibi scintigraphy, and 4-D computerized tomography (4D-CT) [8]. Among these, the most commonly used are US and 99m-Tc sestamibi scintigraphy. Many studies across the globe have shown promising results regarding the sensitivity and specificity of both modalities. However, there are significant differences between the two in terms of cost and availability, with US being less expensive and more readily available.

Pakistan is a developing country with limited healthcare resources, and hence access to modern imaging techniques like 99m-Tc sestamibi scintigraphy is often very difficult. In such a scenario, US of the neck is the imaging modality that is often relied upon for the preoperative localization of parathyroid glands. This study was conducted to evaluate the diagnostic performance of US for the preoperative localization of PHPT at a tertiary care center in Karachi, Pakistan, and compare its diagnostic efficiency with 99m-Tc sestamibi scintigraphy.

## Materials and methods

This retrospective study was conducted at the Aga Khan University Hospital, a tertiary Care Centre in Karachi, Pakistan. It involved patients with PHPT who underwent surgery for PHPT from January 2008 to December 2017. Exemption from ethical approval was obtained from the Ethics Review Committee of the Aga Khan University. Data were retrieved from medical record files of all patients that were coded as “Primary Hyperparathyroidism.” All data were collected in the Health Information Management System of the Aga Khan University Hospital, which was password-protected, and the confidentiality of the patients was fully maintained. Diagnosis of PHPT was made on the basis of hypercalcemia or normocalcemia with high or inappropriately normal serum PTH levels. All patients aged 18 years and above with PHPT who underwent surgery were included. Patients with secondary and tertiary hyperparathyroidism due to end-stage renal disease were excluded. All patients with a known diagnosis of any neoplasia other than parathyroids were also excluded, since in such cases the hypercalcemia could have been related to the malignancy and could not be solely attributed to PHPT.

Preoperative US and 99m-Tc sestamibi scintigraphy findings were recorded and compared to surgical and histological findings, which were taken as the reference standard. The US and 99m-Tc sestamibi scintigraphy were performed by the radiologists at the radiology department of The Aga Khan University Hospital. True positive (TP) refers to cases where abnormality picked on imaging corresponded to the surgical findings. Abnormalities reported by imaging that did not correspond to an abnormal parathyroid gland on surgery were considered false positive (FP). Abnormal parathyroid glands not localized on imaging were recorded as false negative (FN), and negative imaging findings where there was no abnormal parathyroid on surgery were classified as true negative (TN). The sensitivity, positive predictive value (PPV), and accuracy of each of the imaging techniques were then calculated and reported as percentages. MedCalc’s Diagnostic test evaluation online calculator was used to calculate the diagnostic measures.

## Results

A total of 79 patients were identified with PHPT, who underwent surgery from Jan 1, 2008, till Dec 31, 2017. Of the 79 patients, preoperative US was performed in 63 (79.7%) patients and 99m-Tc sestamibi was done in 77 (97.5%). The results of US and 99m-Tc sestamibi scintigraphy and surgical and histological findings of patients were reviewed and analyzed. 

US results were true positive in 53 (84.1%), false positive in three (4.8%), and false negative in seven (11.1%) cases (Table 1). 99m-Tc sestamibi scintigraphy data was true positive in 66 (85.7%), false positive in four (5.2%), and false negative in seven (9.1%) cases (Table 1). There were no true negative cases with either of the modalities (Table 1).

Out of 79 patients, four patients had ectopic parathyroid glands. Among the seven false-negative US cases, four were those who had ectopic parathyroid glands. Among the seven false-negative 99m-Tc sestamibi scans, two were those with ectopic parathyroid glands.

**Table 1:** Description of true positive, false negative, false positive, and true negative cases for ultrasound and 99m-Tc sestamibi scintigraphy

Imaging modality	True positive	False negative	False positive	True negative
Ultrasound neck (n = 63)	53	7 (4 patients had ectopic parathyroid glands. All 4 were not detected)	3 (1 case of papillary thyroid carcinoma)	0
99m-Tc sestamibi scintigraphy (n = 77)	66	7 (4 patients had ectopic parathyroid glands; 2 of them were not detected)	4 (1 case of multinodular goiter)	0

The sensitivity of US was 88.3%, PPV was 94.6%, and accuracy was 84.1% (Table 2). The sensitivity of 99m-Tc sestamibi scintigraphy was 90.4%, PPV was 94.3%, and accuracy was 85.7% (Table 2). The specificity could not be calculated as there were no true negative cases in both modalities.

**Table 2:** Sensitivity, positive predictive value, and accuracy of ultrasound and 99m-Tc sestamibi scintigraphy CI: confidence interval

Imaging modality	Sensitivity [95% CI]	Positive predictive value [95% CI]	Accuracy [95% CI]
Ultrasound neck	88.3% [77.4% – 95.1%]	94.6% [94.2% – 95.0%]	84.1% [72.7% – 92.1%]
99m-Tc sestamibi scintigraphy	90.4% [81.2% – 96.0%]	94.3% [93.9% – 94.7%]	85.7% [75.9% – 92.7%]

## Discussion

US of the neck and 99m-Tc sestamibi are the two most commonly employed and widely studied imaging modalities for the preoperative localization of parathyroids. Different studies have reported a wide range of sensitivity and specificity for them [9-12]. A number of studies have shown their sensitivity and specificity to be comparable; however, the two differ significantly in terms of cost and accessibility [13-14]. Pakistan is a developing country with limited healthcare resources, and modern imaging modalities like 99m-Tc sestamibi are available only in a few big cities. In such a scenario, the US neck seems to be a promising investigative tool, given its diagnostic efficiency, as demonstrated by studies worldwide.

Our current study results (Table 2) are consistent with a recent study by Elsayed and Ali, which reported a US sensitivity of 94.4% and specificity of 44.4%, while the sensitivity and specificity of 99m-Tc sestamibi scintigraphy were 97.4% and 71.4%, respectively [13]. Similar results were seen in a recent study from China, which showed US sensitivity ranging from 90.70% for parathyroid adenoma to 93.5% for parathyroid hyperplasia [15]. Scattergood et al. reported that US had higher sensitivity than 99m-Tc sestamibi scintigraphy [16]. Interestingly, a recent study regarding parathyroid carcinoma localization reported the sensitivity and accuracy of US to be 80% and 73% respectively, of 4-D CT to be 79% and 82%, and of 99m-Tc sestamibi to be 81% and 78% [17].

Very few studies are available regarding PHPT in Pakistan, and the diagnostic performance of US and 99m-Tc sestamibi for preoperative localization of PHPT in our healthcare setup has not been studied [18-20]. There is only one study from Pakistan where preoperative localization of parathyroids with US and 99m-Tc sestamibi was analyzed, and the combined accuracy of both modalities for localization was reported as 100% [20]. However, the individual sensitivity and specificity of the two modalities were not evaluated.

A meta-analysis published in 2017 showed a pooled sensitivity and specificity for US of 80% and 77% respectively, and those for 99m-Tc sestamibi scintigraphy of 83% and 87% respectively [10]. However, evidence in recent years has demonstrated consistent improvement in the sensitivity and specificity of US. In a series reported by Adkisson et al., US was found to be more capable of accurately localizing the parathyroid glands lesion than 99m-Tc sestamibi (63% vs. 41%) [11]. A recent Indian study reported a US sensitivity of 93% [12]. Increasing operator expertise and enhanced technical facilities in the field of imaging appear to have contributed to this improving diagnostic yield, but an additional factor is a rising inclination among endocrine physicians and surgeons to get trained in and perform office-based US.

An audit from Britain reported that the results of surgeon-based US were superior to radiologist-based US in terms of accuracy [21]. A German study demonstrated an overall cure rate of 99.2% with US as the only preoperative localization method when performed by an experienced endocrinologist and/or endocrine surgeon [22]. Similar results were shown by Thomas et al. in a recent study [23]. Another study from Spain also showed comparable results [9].

Ectopic location of the parathyroids in the mediastinum, paraesophageal, and high cervical location or gland weight of less than 200 mg are the common reasons for false-negative US neck. Out of our 79 patients, four were found to have ectopic parathyroids. Out of the seven false-negative US patients in our study, four were those who had ectopically located parathyroid glands in the mediastinum discovered at median sternotomy. Out of the seven false-negative 99m-Tc sestamibi patients, two had ectopically located parathyroid glands in the mediastinum (Table 1). This means that all of the ectopically located parathyroids were not localized by US, whereas 50% of them were localized by 99m-Tc sestamibi. It can be deduced from this information that 99m-Tc sestamibi has a better ability to identify parathyroids in ectopic locations than US.

False-positive US can result from thyroid nodular disease [3,14,24]. Out of the three false-positive US cases, one had papillary thyroid carcinoma found on exploration. False-positive 99m-Tc sestamibi scans have been attributed to thyroid adenomas, lymph nodes, diffuse hyperplasia, and metastatic thyroid cancer. Out of our four false-positive 99m-Tc sestamibi scans, one had multinodular goiter (Table 1).

While practicing in a resource-constrained healthcare system, it is the paramount responsibility of the physician to take into account the cost and accessibility of any investigation before ordering them. In Pakistan, the average cost of a parathyroid US is approximately $40, whereas the average cost of 99m-Tc sestamibi is approximately $155. This means that the cost of 99m-Tc sestamibi is almost four times higher than that of US. This amount would further increase significantly if the cost of traveling and other logistics are taken into account since the facility of 99m-Tc sestamibi is available only in a few big cities of the country. Evidence has shown that using US as the principal imaging modality and limiting 99m-Tc sestamibi to only those cases where US fails to localize the lesion could decrease the cost of imaging by 53% [25]. This approach appears to be a reasonable way of increasing the cost advantage of imaging in a resource-constrained healthcare system of a developing country like Pakistan.

## Conclusions

US neck is an efficient tool for preoperative localization of PHPT, demonstrating a comparable diagnostic efficiency with 99m-Tc sestamibi, and can serve as a credible first-line imaging modality in a resource-constrained healthcare setup. A reasonable and cost-effective approach is to limit the use of 99m-Tc sestamibi to only those cases where US fails to localize the lesion. The practice of office-based US has currently not been adopted in Pakistan. Endocrinologists and endocrine surgeons in Pakistan should be encouraged to develop expertise in performing office-based US, which can further increase diagnostic efficiency, accessibility, and cost-effectiveness of imaging.
